# Determinants of nutritional status and outcome in adults with RCSE: a retrospective cohort study

**DOI:** 10.1186/s12883-021-02373-8

**Published:** 2021-09-08

**Authors:** Zhang Yu, Liu Ling

**Affiliations:** 1 Department of Neurology, West China Hospital, Sichuan University, Wai Nan Guo Xue Lane 37 #, 610041 Chengdu, Sichuan China; 2 Department of Neurology, Chengdu Shangjin Nanfu Hospital, Shang Jin Road 253#, 610000 Chengdu, Sichuan China

**Keywords:** Refractory convulsive status epilepticus, Nutritional characteristics, Association

## Abstract

**Background:**

This study aimed to investigate the association between nutritional characteristics in patients with refractory convulsive status epilepticus.

**Methods:**

We retrospectively enrolled 73 patients with refractory convulsive status epilepticus over 18 years of age at the West China Hospital between January 2017 and May 2019. All patients met the 2016 International League Against Epilepsy diagnostic criteria for refractory convulsive status epilepticus. A logistic regression model was used to evaluate the association between malnutrition and refractory convulsive status epilepticus.

**Results:**

Of the 73 patients with refractory convulsive status epilepticus, 33 (45.21 %) suffered from malnutrition during hospitalization, and duration of hospitalization in days (OR = 1.251; 95 % CI,–1.067–1.384; P = 0.007), nasal feeding (OR = 22.623; 95 % CI: 1.091-286.899; *P* = 0.013), and malnutrition on admission (OR = 30.760; 95 % CI: 1.064–89.797; P = 0.046) were significantly associated with malnutrition in patients with refractory convulsive status epilepticus.

**Conclusions:**

Malnutrition is a common complication during hospitalization in patients with refractory convulsive status epilepticus. The duration of hospitalization (days), nasal feeding, and malnutrition at admission are associated with malnutrition in patients with refractory convulsive status epilepticus. Further longitudinal studies are needed to identify the relationship between refractory convulsive status epilepticus and adverse outcomes.

## Background

Convulsive status epilepticus is one of the most common critical and severe neurological disorders [1–3]. Its main feature is continuous epileptic seizures, with high disability and death rates, while refractory convulsive status epilepticus (RCSE) has higher complication and mortality rates [2, 4, 5]. Refractory convulsive status epilepticus, which accounts for 23–48 % of convulsive status epilepticus, refers to a state in which sufficient doses of first-line antiepileptic drugs (such as benzodiazepines) and subsequent intravenous antiepileptic drugs are unable to terminate a seizure, and the electroencephalogram (EEG) shows pathological discharge [2, 4, 5]. The fatality rate is as high as 23–61 %, which is three times higher than that of non-refractory convulsive status epilepticus [2, 4–6]. Malnutrition is a common complication that occurs during hospitalization of patients with RCSE, and might be associated with a poor prognosis. Malnutrition is usually defined as malnutrition caused by insufficient intake, poor absorption, or excessive loss of nutrients, resulting in physical and mental functional defects [7]. Many studies have shown that the nutritional status of patients suffering from RCSE deteriorates continuously during hospitalization. Despite receiving nutritional support, various nutritional parameters remain on a downward trend, and this undernutrition could be associated with the poor prognosis of patients [7–11]. Rybitschka et al. study reveals independent associations between nutrition support and outcome ,and increased calorie intake during SE is independently associated with unfavorable outcome in adult SE patients [12]. But malnutrition in patients with RCSE during hospitalization has not yet been reported. The nutritional status of the patients was evaluated at the time of admission, and it may be important for the medical staff to identify the risk of malnutrition as soon as possible. Therefore, this study aimed to explore the association and prognosis of nutritional characteristics in patients with RCSE during hospitalization to develop strategies and purposeful early interventions and to improve the prognosis of these patients.

## Methods

### Study design

This was a retrospective study that included 73 RCSE patients from the NICU, neurology, and other departments in the West China Hospital from January 2017 to May 2019. All patients met the latest diagnostic criteria of the International League Against Epilepsy (ILAE) [1, 13]. According to the standards of the Declaration of Helsinki, this study strictly abided by the principles of patient willingness and knowledge. If the patient did not have conscious autonomy, the willingness and knowledge of his immediate family members were obtained.

Inclusion criteria included age ≥ 18 years; conformed to the latest diagnostic standard of the ILAE for RCSE [1]; treated according to the American Epilepsy Society (AES) 2016 Status Epilepticus Guidelines [13]; and voluntary and informed consent.

Exclusion criteria included:primary psychosis or serious anxiety and depressive disorder; liver cirrhosis, renal insufficiency, and other conditions that require limiting protein intake; hospitalization for over less than 2 weeks; and patients with no available EEG.

### Research methods

Based on the inclusion and exclusion criteria, general patient data (including demographic information, hospitalization time, and expenses), serum albumin detected within 36 h of admission and at the time of discharge, height and weight, body mass index (BMI), and clinical characteristics at admission (including altered consciousness, dysphagia, diet reduction, stress ulcer, and nutritional status) were collected. All RCSE patients were treated in accordance with the American Epilepsy Society (AES) 2016 Status Epilepticus Guidelines [14].

To evaluation dysphagia, the patient was instructed to drink 30 ml of water in sitting position. The results were classified as follows: Grade 1, smooth swallowing with no choking coughs or pauses; Grade 2, swallowing twice without choking coughs or pauses, or prolongation for more than 5 s; Grade 3, capable of swallowing at once but with a choking cough; Grade 4, more than two attempts at swallowing but still with a choking cough; and Grade 5, a frequent choking cough with a full swallow being difficult [15, 16]. Dysphagia was suspected in the presence of symptoms of dysphagia, and if the drinking water test was ≥ grade 3 or there was a altered consciousness [15] Nutritional Assessment Form was completed. The Glasgow coma score was used to evaluate the level of consciousness.

### Nutritional status assessment

Nutritional status assessments were carried out by specialized personnel 24 h after admission and discharge, including measurements of body weight and height and calculation of the BMI (BMI = body weight [kg] /height [m^2^] ). The indexes of serum albumin, serum transferrin, and serum prealbumin were determined. The Mini-Nutritional Assessment Form was completed [17]. Malnutrition was diagnosed if the patient met at least one of the following four criteria: (1) BMI < 18.5 kg/m^2^; (2) serum albumin concentration < 35 g/l; (3) weight loss at discharge greater than 6 % of the weight at admission; and (4) MNA < 17. An MNA score of 17–23.5 indicated the risk of malnutrition [7, 8].

Nutritional support includes parenteral nutrition (PN) and enteral nutrition (EN). PN was defined as the intravenous infusion of fat emulsions, amino acids, and glucose, which includes a single bottle infusion of amino acids or fat emulsions. EN included tube feeding and oral nutritional supplementation.

### Data statistics and analysis

Continuous data were presented as the mean and standard deviation, while categorical data were presented as frequencies and percentages. Fisher’s exact test and the chi-square test were used to compare proportions between categorical variables, while Student’s t-test was used to compare proportions between continuous variables. Univariate analysis was performed, followed by multivariate analysis of the variables that showed significant associations during univariate analysis. The threshold for statistical significance was set at *P* < 0.05. We used SPSS version 22.0 for statistical analysis.

## Results

A total of 33 out of 73 patients with RCSE were malnourished. There were no significant differences between the demographic characteristics of the 33 malnourished patients (16 men, mean age 39 ± 21 years) and the 40 patients who were not malnourished (28 men, aged 40 ± 20 years). Patients suffering from malnutrition had a significantly longer duration of hospitalization, higher hospitalization expenses, and significantly lower body weight and serum albumin when discharged from the hospital than patients the ones who were not malnourished (Table 1).

**Table 1:** Demographic and clinical data of patients included. (CNS: Central nervous system)

	Total (73)	Malnutrition (33/45.2 %)
Sex(male,%)	44(60.3 %)	16(21.9 %)
Age(years,range)	40 ± 20	39 ± 21
Hospital stays(day)	44 ± 23	82 ± 49
Cost(thousand dollar)	149,611 ± 314,605	303,997 ± 420,872
BMI (kg /m2)	55 ± 30	50 ± 25
Albumin(g / L)	41 ± 10	30 ± 7
Pathogen		
CNS-infection acute	30	15
Head trauma	3	1
Brain tumor	7	4
CNS-anomalies	7	2
cerebrovascular disease	5	2
Anoxia	4	1
Metabolic sodium balance and metabolic disorder	6	4
Drug reduction/withdrawal,poor compliance	11	4
Malnutrition on admission	7	5
Diabetes mellitus	11	5
Tube feeding	31	28
Reduced level of consciousness	25	23
Depressed mood	8	4
Pneumonia and infection	48	30
Tumor	6	3
Gastrointestinal bleeding	15	11
Dementia	3	1
Ketone diet	1	1
Dysphagia	4	4
Need for tracheal intubation	27	26

The causes of 73 cases of RCSE included acute central nervous system infection (30 cases), head trauma (3 cases), brain tumor (7 cases), acute cerebrovascular disease (5 cases), ischemic anoxic encephalopathy (4 cases), other central nervous system abnormalities (7 cases), electrolyte/acid-base imbalance (6 cases), and poor drug compliance/withdrawal (11 cases). In 11 RSE patients with drug compliance/withdrawal, epilepsy averaged 2–12 years. The following drugs were administered: sodium valproate (2 patients), oxcarbazepine (1 patient), levetiracetam (3 patients), topiramate (1 patient), lamotrigine (1 patient), and levetiracetam and sodium valproate (3 patients). The causes of RCSE in the 33 cases of malnutrition included central nervous system infection (15 cases), head trauma (1 case), brain tumor (4 cases), acute cerebrovascular disease (2 cases), ischemic anoxic encephalopathy (1 case), other central nervous system abnormalities (2 cases), electrolyte acid-base imbalance (4 cases), and poor drug compliance/withdrawal (4 cases) (Table 1).

Upon admission, 7 patients (9.59 %) had malnutrition: 4 with BMI < 18.5 kg/m^2^ and 3 with serum albumin < 35 g/l. There were 33 malnourished patients (45.21 %) at the time of discharge, including 15 patients with weight loss greater than 6 % of their body weight at the time of admission, 18 patients with BMI < 18.5 kg/m^2^, 19 patients with serum albumin < 35 g/l, 33 patients with MNA < 17 points, and 11 patients with MNA < 17–23.5 points. The incidence of malnutrition at discharge was significantly higher than that at admission (*p* < 0.05). The ratio of serum albumin < 35 g/l at discharge was significantly higher than that at admission (*p* < 0.01) (Table 2).

**Table 2:** Nutritional Status of Stroke Patients at Different Time Points of Admission

	Admission	7 day	14 day	Discharge
Weight loss is greater than 6 % of the body weight at admission	0	2	10	15
BMI < 18.5 kg /m2	4	8	14	18
Albumin < 35 g / L	3	9	13	19
MNA < 17	2	9	18	33

Multivariate logistic regression analysis was conducted on the collected data to obtain values for the duration of hospitalization in days (OR = 1.251; 95 % CI: 1.067–1.384; *P* = 0.007), nasal feeding (OR = 22.623; 95 % CI: 1.091–286.899; *P* = 0.013), and malnutrition on admission (OR = 30.760; 95 % CI: 1.064–89.797; *P* = 0.046), which were significantly associated with malnutrition in RCSE. However, sex, age, mechanical ventilation, tumor, decreased consciousness level during hospitalization, tumor, pneumonia, dementia, ketogenic diet, gastrointestinal hemorrhage, diabetes, depression, and dysphagia all had *P*-values > 0.05 (Table 3).

**Table 3:** Risk factors for malnutrition in patients with refractory status epilepticus

	Total(73)	*P*-value	OR	95 %CI
Male	44(60.3 %)	0.529	0.189	0.002–17.306
Age	40 ± 20	0.356	1.058	0.938–1.098
Hospital stays(day)	44 ± 23	0.007	1.251	1.067–1.384
Malnutrition on admission	7	0.042	30.760	1.064–89.797
Diabetes mellitus	11	0.734	2.314	0.018-234.998
Tube feeding	31	0.013	22.623	1.091-286.899
Reduced level of consciousness	25	0.137	0.048	0.001–2.645
Depressed mood	8	0.695	0.504	0.017–15.394
Pneumonia and infection	48	0.629	3.180	0.111–7.218
Tumor	6	0.664	0.472	0.015–14.865
Gastrointestinal bleeding	15	0.750	2.334	0.013-433.271
Dysphagia	4	0.079	29.133	0.680-456.372
Need for tracheal intubation	27	0.320	22.336	0.025-197.623

Ten patients died upon discharge from the hospital, and 17 patients had MRS scores > 2. Multivariate logistic regression analysis showed that patients with malnutrition or risk of malnutrition had a worse prognosis than those with good nutritional states (*p* < 0.05).

## Discussion

During hospitalization, although RCSE patients received nutritional support, various nutritional parameters showed a downward trend. Malnutrition rates were 9.59 % at the time of admission and 45.21 % at the time of discharge. Currently, there is no universally accepted definition of malnutrition, and there is no gold standard for nutritional assessment. Previous studies identified serum albumin as the most significant indicator of a person’s nutritional status, and this parameter is often used; however, its half-life is relatively long, approximately 14–20 days [18, 19]. In addition, the use of a variety of nutritional assessment tools, such as the MNA, could help to make a broad estimate of malnutrition [17]. There are many reasons for malnutrition during hospitalization, which are related to insufficient intake, gastrointestinal tract malabsorption, and increased energy consumption.

In RCSE patients who fast due to a altered consciousness, gastrointestinal hemodynamics are affected due to inadequate food stimulation, and gastrointestinal digestion and absorption functions weaken, or even reduce to zero, which culminates in malnutrition [7, 20]. When gastrointestinal dynamics are weakened, an increase in gastrointestinal pressure could occur, coupled with damage to the gastrointestinal mucosal structure caused by ischemia. A large amount of bacteria and toxins in the gastrointestinal tract can enter the blood, causing systemic infection and poisoning symptoms, aggravating the body’s energy consumption, eventually leading to malnutrition [20]. In this study, the level of consciousness of patients in the malnutrition group decreased more significantly after admission; the functional status of daily activities was lower, and there were more gastrointestinal complications, such as pulmonary infection, heart failure, electrolyte disturbance, and poor gastrointestinal function.

This study shows that malnutrition on admission, nasal feeding, and lengthy hospitalization are associated with malnutrition during hospitalization in patients with RCSE. Malnutrition on admission is the basis of malnutrition, which indicates that pneumonia, other infections, gastrointestinal hemorrhage, and other related conditions are more likely to occur during hospitalization, leading to decreased food consumption in patients, which results in malnutrition. The longer the hospital stay, the more likely patients are to have nosocomial infections and complications (such as bedsores and gastrointestinal hemorrhage), and the nutritional intake of patients during hospitalization is worse than that at admission. The diet accepted by nasal-feeding patients is mainly a liquid diet, such as protein powder and rice flour, which is associated with a high risk of diarrhea.

Previous studies have shown that dysphagia is associated with malnutrition after stroke, which is inconsistent with the conclusions of this study [15, 16, 21–24]. The reason for this difference may be that only 5 patients with RCSE caused by cerebral infarction were included in this study, and the study sample was small. More patients with cerebral infarction could be included in a follow-up study, and the sample size could be expanded and analyzed separately. Decreased consciousness levels in patients during hospitalization could also be associated with malnutrition in patients with refractory epilepsy. When a patient’s consciousness level decreases, nutritional intake might decrease, and decreased intestinal peristalsis during long-term bed rest may lead to poor absorption. However, the results of this study did not enable us to arrive at this conclusion, which may be because the included patients were seriously ill, and most of them had decreased levels of consciousness.

In addition to malnutrition, nasal feeding, and the duration of hospitalization shown in this study, there may be other related factors. These factors include (1) concomitant consumptive diseases; (2) some patients with intractable convulsive epilepsy continued to use a ketogenic diet, mainly a high-fat diet lacking protein involvement; (3) severe complications, such as upper gastrointestinal hemorrhage, respiratory and urinary tract infection, multiple organ failure, bedsores, and dementia, increase the body’s energy consumption; and (4) depression and other mental disorders can lower patients’ self-confidence, resulting in anorexia, non-cooperation with treatment, and other manifestations, which predisposes them to malnutrition. There are also (5) iatrogenic factors–some doctors pay one-sided attention to the role of clinical drug therapy, ignoring nutritional support.

This study shows that the prognosis of malnourished patients is usually poor; these patients may be more prone to nosocomial infections, gastrointestinal dysfunction, and other complications that may have occurred accidentally due to the small sample size or due to the limitations of our evaluation tools.

This study has some limitations. First, the data we analyzed and reported were obtained from the West China Hospital. Although they may reflect an association between malnutrition and RCSE in patients in western China to some extent, their generalizability is poor because it was a single-center study. At present, there is no universally accepted definition of malnutrition and no gold standard for nutritional assessment. Thus, the research results have certain limitations. In subsequent research, the sample size will be further expanded, and etiology-based subgroup analysis will be conducted. We also regret that we did not measure micronutrients, which means we do not know if there were micronutrient deficiencies. Only patients over 18 years of age were included in this study; these patients may have some differences from those in other age groups had they been included in this study.

## Conclusions

The Prevalencerisk of malnutrition during hospitalization in patients with RCSE was 45.21 %. Malnutrition at admission, nasal feeding, and the duration of hospitalization (days) were associated with malnutrition. Patients who were malnourished on admission are more likely to develop malnutrition during hospitalization, and early intervention is required for these patients.

## Data Availability

The data generated and analyzed during the current study are not publicly available because of the lack of consent from all patients; however, they are available from the corresponding author upon reasonable request.

## Abbreviations

RCSE: Refractory convulsive status epilepticus
ILAE: International League Against Epilepsy
AES: American Epilepsy Society
EEG: Electroencephalogram
NICU: Neurology intensive care unit
BMI: Body mass index
MNA: Mini Nutrition Assessment

## References

[1] Trinka E, Cock H, Hesdorffer D, Rossetti AO, Scheffer IE, Shinnar S (2015). A definition and classification of status epilepticus - report of the ILAE Task Force on Classification of Status Epilepticus. Epilepsia.

[2] Koubeissi M, Alshekhlee A (2007). In-hospital mortality of generalized convulsive status epilepticus. Neurology.

[3] Cederholm T, Barazzoni R, Austin P, Ballmer P, BIolo G, Bischoff SC (2017). ESPEN guidelines on definitions and terminology of clinical nutrition. Clin Nutr.

[4] Hocker S, Britton JW, Mandrekar JN, Wijdicks EFM, Rabinstein AA (2013). Predictors of outcome in refractory status epilepticus. JAMA Neurol.

[5] White JV, Guenter P, Jensen G, Malone A, Schofield M (2012). Consensus statement of the Academy of Nutrition and Dietetics/American Society for Parenteral and Enteral Nutrition: characteristics recommended for the identification and documentation of adult malnutrition (undernutrition). J Acad Nutr Diet.

[6] Lai A, Outin H, Jabot J, Megarbane B, Gaudry S, Coudroy R (2015). Functional outcome of prolonged refractory status epilepticus. Crit Care.

[7] Suter PM (2004). Alcohol, nutrition and health maintenance: selected aspects. Proc Nutr Soc.

[8] Lipkin EW, Bell S (1993). Assessment of nutritional status: the clinician’s perspective [J]. Clin Lab Med.

[9] MacIntosh C, Morley JE, Chapman IM (2000). The anorexia of aging[J]. Nutrition.

[10] Rybitschka A, Semmlack S, Kaplan PW, De Marchis GM, Rüegg S, Marsch S, Sutter R. Calorie Intake During Status Epilepticus and Outcome: A 5-Year Cohort Study. Crit Care Med. 2019 Aug;47(8):1106–1115. doi: 10.1097/CCM.0000000000003828. PMID: 31135501. 10.1097/CCM.0000000000003828 31135501

[11] Glauser T, Shinnar S, Gloss D, Allredge B, Arya R, Bainbridge J (2016). Evidence-based guideline: Treatment of convulsive status epilepticus in children and adults: Report of the Guideline Committee of the American Epilepsy Society[J]. Epilepsy Curr.

[12] Davalos A, Ricart W, Gonzalez-Huix F, Soler S, Marrugat J, Molins A (1996). Effect of malnutrition after acute stroke on clinical outcome. Stroke.

[13] Madžar D, Geyer A, Knappe RU, Gollwitzer S, Kuramatsu JB, Gerner ST (2016). Association of seizure duration and outcome in refractory status epilepticus. J Neurol.

[14] Rosenow F, Hamer HM, Knake S (2007). The epidemiology of convulsive and nonconvulsive status epilepticus. Epilepsia.

[15] Feigin VL, Forouzanfar MH, Krishnamurthi R, Mensah AG, Connor M, Bennet DA (2014). Global and regional burden of stroke during 1990–2010: findings from the global burden of disease study 2010. Lancet.

[16] Gomes F, Emery PW, Weekes CE (2016). Risk of malnutrition is an independent predictor of mortality, length of hospital stay, and hospitalization costs in stroke patients. [J] Stroke Cerebrovasc Dis Off J Nat Stroke Assoc.

[17] Aquilani R, Sessarego P, Iadarola P, Barbieri A, Boschi F (2011). Nutrition for brain recovery after ischemic stroke: an added value to rehabilitation. Nutr Clin Pract.

[18] Leitinger M, Holler Y, Kalss G, Rohracher A, Novak HF, Hofler J (2015). Epidemiology-based mortality scale in status epilepticus (EMSE). Neurocrit Care.

[19] Zhao YY, Zeng W (2016). Nutritional status and influencing factors of malnutrition in elderly patients with stroke. Chin J Gerontol.

[20] Guigoz Y, Vellas BJ, Garry PJ (1994). Mini Nutritional Assessment: a practical assessment tool for grading the nutritional state of elderly patients [J]. Facts ï¼²es Gerontonol.

[21] Elia M, Stroud M (2004). Nutrition in acute care[J]. Clin Med.

[22] Donini LM, Savina C, Cannella C (2003). Eating habits and appetite control in the elderly: the anorexia of aging [J]. Int Psychogeriatr.

[23] Donini LM, De Bernardini L, De Felice MR, Savina C, Coletti C, Cannella C (2004). Effect of nutritional status on clinical outcome in a population of geriatric rehabilitation patients [J]. Aging Clin Exp ï¼²es.

[24] Wang W, Jiang B, Sun H, Xiaojuan R, Donglin S, Linhong W (2017). Prevalence, incidence, and mortality of stroke in China: results from a nationwide population-based survey of 480,687 adults. Circulation.

